# Effectiveness and safety of massage in the treatment of obesity: A protocol for systematic review and meta-analysis

**DOI:** 10.1097/MD.0000000000025249

**Published:** 2021-03-26

**Authors:** Mengke Jin, Lin Jiao, Jun Li, Daocheng Zhu, Wei Xu, Genping Zhong, Zhiwen Cao, Xuefang Liu

**Affiliations:** a Jiangxi University of Traditional Chinese Medicine; b The Affiliated Hospital of Jiangxi University of Traditional Chinese Medicine, Nanchang, China.

**Keywords:** massage, obesity, protocol, systematic review

## Abstract

**Background:**

Obesity has become one of the largest chronic diseases in the world. It is a chronic metabolic disease caused by various factors. In recent years, massage has been used more and more widely in the treatment of obesity diseases. However, the effectiveness and safety of massage in the treatment of adult obesity are still unclear. The purpose of this study is to evaluate the effectiveness and safety of massage in the treatment of adult obesity.

**Methods:**

We will conduct a comprehensive review in Medline, PubMed, Cochrane System Evaluation Database, embbase, Chinese Biomedical Literature Database, China National Knowledge Infrastructure, Wang Fang Database, Chinese Science Journal Database. There is no language restriction for the literature search from its establishment to February 2021. In addition, we will manually search for references to unpublished studies and originally included articles. Reviewers will identify the research, extract the data, and independently assess the quality. Results of interest include: total effective rate; total nasal symptom score; rhinitis quality-of-life questionnaire; visual analog scale; laboratory test indicators: IgE, IL6, IL10, or TNF-α levels; recurrence rate; adverse events. Randomized clinical trials will be collected, the Cochrane bias risk assessment tool will be used to assess methodological quality, and recommendations, evaluation, development, and evaluation methods will be used to assess the level of evidence. The meta-analysis will be performed using RevMan 5.4.0 software. A heterogeneity test will be conducted between studies, and *P* <.1 and I2> 50% are the thresholds for testing. According to the degree of heterogeneity, we will use a fixed effects model or a random effects model.

**Results:**

The results of this study will provide sufficient evidence to judge whether massage is an effective and safe treatment for adult obesity.

**Conclusions:**

This study will provide evidence to determine whether massage is an effective intervention for Adult obesity. The research results will also be published in a peer-reviewed journal.

**INPLASY registration number:**

INPLASY 202120061

## 1 Introduction

Obesity is a chronic and complex disease. It is characterized by excessive accumulation of adipose tissue, leading to a significant increase in body weight.^[1]^ As early as 1948, it was included in the list of diseases by the World Health Organization.^[2]^ At present, the disease has become one of the largest chronic diseases in the world. The World Health Organization defines obesity as: excessive fat accumulation that may damage health, pointing out that when body mass index (BMI) ≥ 24.9 kg/m^2^ is overweight; when BMI ≥ 30 kg/m^2^, it can be diagnosed as obesity; when the patient exceeds 40 kg/m^2^ is extremely severely obese.^[3]^ Its formation is mainly due to the imbalance between total calorie intake and expenditure. The intake is greater than the expenditure, which leads to excessive accumulation of fat in the body, which leads to obesity.^[4]^ From an evolutionary perspective, human ancestors often endured hunger due to insufficient food in the early harsh environment. Under this condition, once they had food, their bodies would quickly store and absorb the energy in the food to prevent them from falling Hunger. Under the influence of this environment, it led to the formation of a genotype prone to overeating, low-energy consumption, and lack of exercise. Compared with people who are not hungry, human beings who can endure hunger for a long time and can store and mobilize energy more effectively have stronger survivability and reproductive capabilities. This will lead to genetic variation in the population. The main manifestation is that these people eat faster and have a higher ability to absorb calories. This in turn makes the energy storage in adipose tissue more efficient. Under the development of this evolutionary trend, in recent years, the proportion of obese people has been increasing.^[5]^ In the past 50 years, the obesity prevalence has increased worldwide to the pandemic ratio.^[6]^ Data research shows that from 1980 to 2013, the proportion of overweight adults in developed countries increased by 28%, while the proportion of overweight adults in developing countries increased by nearly 60%.^[7]^ Between 1975 and 2014, the global prevalence of obesity in adult men (BMI≥30 kg/m^2^) rose from 3.2% to 10.8%; the prevalence of obesity in adult women rose from 6.4% to 14.9%.^[8]^ From 1990 to 2015, the obesity rate in 73 countries doubled, and the obesity rate in other countries also continued to rise.^[9]^ A survey conducted by the World Health Organization in 2017 showed that at present, more than 2 billion adults in the world are overweight, and more than 650 million adults are obese. In the future, the number will continue to increase for several decades.^[4,10]^

Obesity is caused by various factors such as genetic factors and environmental factors. Relevant studies have shown that obesity has become a risk factor for type 2 diabetes, cardiovascular disease, high blood pressure, stroke, and various cancers. The emergence of obesity and its related complications will affect the work and life of patients. In severe cases, it will also affect social and economic development and cause medical and economic burdens to the country and society.^[6]^ Studies in European and American countries have shown that the burden of disease caused by obesity accounts for 3% to 7% of the total national medical expenditure, of which as high as 10% in the United States.^[11]^ The number of deaths due to obesity in the United States each year exceeds 300,000, ranking second in preventable deaths, and the annual expenditure on obesity treatment exceeds 100 billion US dollars.^[12]^ The annual cost of medical treatment for obese patients is more than that of nonobesity is $1492 higher.^[13]^

Obesity has an increasing impact on individuals, families, and society. Therefore, the treatment of obesity is becoming more and more important. Western medicine currently treats obesity mainly in 2 ways: drug treatment and surgical treatment. Surgical treatment mainly includes gastric ligation (gastric ligation), liposuction, and so on. However, it should be noted that the applicable population of the surgical method is mainly severely obese and extremely obese patients, and it is not suitable for ordinary patients.^[14,15]^ Surgical therapy also has many disadvantages, such as great harm to the body, high risk, and high cost. At present, the most commonly used method in Western medicine is still medication. Mainly include orlistat, phentermine, bupropion, bupropion sustained-release agent. In addition, it also includes some hypoglycemic drugs that also have a weight loss effect.^[15]^ Orlistat inhibits the lipase in the gastrointestinal tract, prevents the hydrolysis of triacylglycerols into free fatty acids and monoacylglycerol ester, reduces the absorption of fat by the intestinal mucosa, and promotes the elimination of fat from the body, thereby achieving the effect of weight loss. However, the drug is prone to some gastrointestinal reactions, such as increased gastrointestinal gas, fatty stool, fecal incontinence, rectal pain, and so on. Phentermine was approved for short-term (3 months) weight loss treatment in 1960.^[16]^ But this type of drug has many adverse reactions, such as increased blood pressure, insomnia, and teratogenicity.

Bupropion sustained release agents consist of opioid receptor antagonists and dopamine and norepinephrine reuptake inhibitors. It reduces appetite by affecting the dopamine pathway in the feeding center or limbic system of the hypothalamus to lose weight. However, the drug can easily cause adverse reactions such as dry mouth, insomnia, dizziness, headache, nausea, and vomiting.^[15]^

In addition, it also includes some dietary adjustment methods. Mainly include very low calorie diet therapy, low carbohydrate diet and High-protein diet, etc. Very calorie diet therapy has obvious weight loss effects in the short term, but it is easy to cause headache, dizziness, and water and electrolyte disturbances.^[17]^ Low carbohydrate diet is also known as the “ketogenic diet.” This method is only effective in a short period of time, and it is prone to dizziness, headaches, muscle cramps, and other phenomena.^[18]^ High-protein diet means that the daily protein intake exceeds 20% of the total energy or is above 1.5 g/kg.^[19]^ Although these drugs have obvious weight loss effects, they can easily cause kidney damage.

The word obesity first appeared in the Warring States Period.^[20]^ The book “Spleen and Stomach Theory” believes that obesity is caused by eating too much greasy food, which in turn will breed phlegm. The book divides obesity into 2 pathogenesis. With strong spleen and stomach functions, people will have a strong appetite, so they will eat a lot of food and then become fat. The patient's spleen and stomach are weak, the food ingested cannot be digested and absorbed, and it stays in the intestines and stomach to form fat. The spleen is weak and the limbs cannot get nourishment. Therefore, the main feature of this obesity is the body is obese, but the limbs are weak.^[21]^ Li et al^[22]^ believe that the main cause and pathogenesis of obesity is insufficient kidney qi, which cannot promote the transformation of qi in the bladder, leading to excessive sputum, which leads to obesity. In addition, obesity is also affected by factors such as emotions and lack of exercise.^[23]^

There are many ways to treat obesity in Chinese medicine. These mainly include acupuncture, acupoint embedding, massage, and other treatment methods.^[20]^ In recent years, massage therapy has been used more and more in the treatment of obesity. Many literature reports have also pointed out that massage has obvious effects in the treatment of obesity and the decomposition of gluteal muscle adipose tissue.^[24,25]^ There are also related literatures on the treatment of simple obesity by massage, but there is no literature on the treatment of all types of obesity in adults. Therefore, this study comprehensively evaluates the effectiveness and safety of Tuina in the treatment of adult obesity through systematic reviews and meta-analysis.

## 2 Methods

### 2.1 Study registration

This system evaluation plan has been registered on the International Prospective Register of Systematic Reviews (registration numberINPLASY202120061). You can check its authenticity on this website https://inplasy.com/inplasy-2021-2-0061/). This article does not require ethical approval. The agreement follows the “Cochrane System Evaluation and Meta-Analysis Protocol Manual.”

### 2.2 Criteria for inclusion

#### 2.2.1 Types of studies

The study will include randomized controlled trials of all massage-related therapies related to obesity, regardless of language used. However, nonrandomized controlled trials, animal trials, case studies, research progress, expert experience, conference articles, and some duplicated articles will be excluded.

#### 2.2.2 Types of participant

The subjects of this study are all adults who have been diagnosed with obesity, without gender and race restrictions.

#### 2.2.3 Types of interventions

The intervention measures of the experimental group mainly include any form of massage therapy for obesity treatment, which includes traditional Chinese massage, massage, acupoint massage, therapeutic massage, full body massage, acupoint massage, relaxation, and so on. But massage combined with other interventions, such as acupuncture, moxibustion, functional exercise, and other mixed therapies, will be excluded.

#### 2.2.4 Control interventions

The control group includes any form of treatment, as long as it is an internationally recognized method, such as acupuncture and Chinese medicine. In addition, it also includes nonintervention and placebo. However, we will exclude comparative studies between the efficacy of different types of massage techniques.

#### 2.2.5 Types of outcome measurements

##### 2.2.5.1 Primary outcome

These mainly include clinical symptoms, BMI and body weight index reduction.

##### 2.2.5.2 Additional outcomes

1. Reduction in the following data: waist circumference, waist–hip ratios, body fat mass percent, body fat mass, serum cholesterol, triglyceride, low-density lipoprotein cholesterol.
2. Elevated high-density lipoprotein cholesterol.
3. The incidence rate of adverse events.

#### 2.2.6 Exclusion criteria

1. In the experimental group, those without massage techniques will be excluded.
2. Review categories, nonrandom trials, animal reports, pathology reports, etc will be excluded.
3. Randomized controlled trials that compare the effects of massage manipulation on obesity treatment will also be excluded.
4. Documents with unclear information will be excluded.

### 2.3 Search methods for identification of studies

#### 2.3.1 Electronic data sources

The search content is that the database is established until February 2021. We will search from the following 4 English databases: PubMed, EMBASE, Cochrane Central Register of Controlled Trials, Web of Science. Chinese data retrieval will be conducted from the following Chinese databases: China Biomedical Literature Database, Wan fang Database, Chongqing VIP Database (VIP), and China Knowledge Network. The specific PubMed retrieval strategy is shown in Table 1. The search strategies of major databases will be adjusted according to different databases.

**Table 1:** The search strategy for PubMed.

Order	Strategy
#1	Search: “Obesity”[Mesh] or “Obesity, Abdominal”[Mesh] or “Obesity, Morbid”[Mesh] or “Obesity Management”[Mesh] or “Obesity, Metabolically Benign”[Mesh]
#2	Search: “Abdominal Obesities” [Ti/Ab] or “Obesities, Abdominal” [Ti/Ab] or “Abdominal Obesity” [Ti/Ab] or “Central Obesity” [Ti/Ab] or “Central Obesities” [Ti/Ab] or “Obesities, Central”
#3	#1 OR #2
#4	Search: “Massage”[Mesh] OR “Musculoskeletal Manipulations”[Mesh]
#5	Search: “Zone Therapy”[Ti/Ab] or “Therapies, Zone ” [Ti/Ab] or “Therapies, Zone” [Ti/Ab] or “Tuina”[Ti/Ab] or “Zone Therapies” [Ti/Ab]
#6	#4 OR#5
#7	Search: “Adult: 19+ y”
#8	Search: “Adults” [Title/Abstract]
#9	#7 OR#8
#10	Search: “Randomized controlled trial” [MeSH] or “controlled clinical trial” [MeSH]
#11	Search: “Randomized controlled trial” [Ti/Ab] or “clinical trial” [Ti/Ab] or “randomized” [Title/Abstract]
#12	#10 OR #11
#10	#3 AND #6 AND #9 AND #12

### 2.4 Data collection

#### 2.4.1 Selection of studies

**Figure 1:**
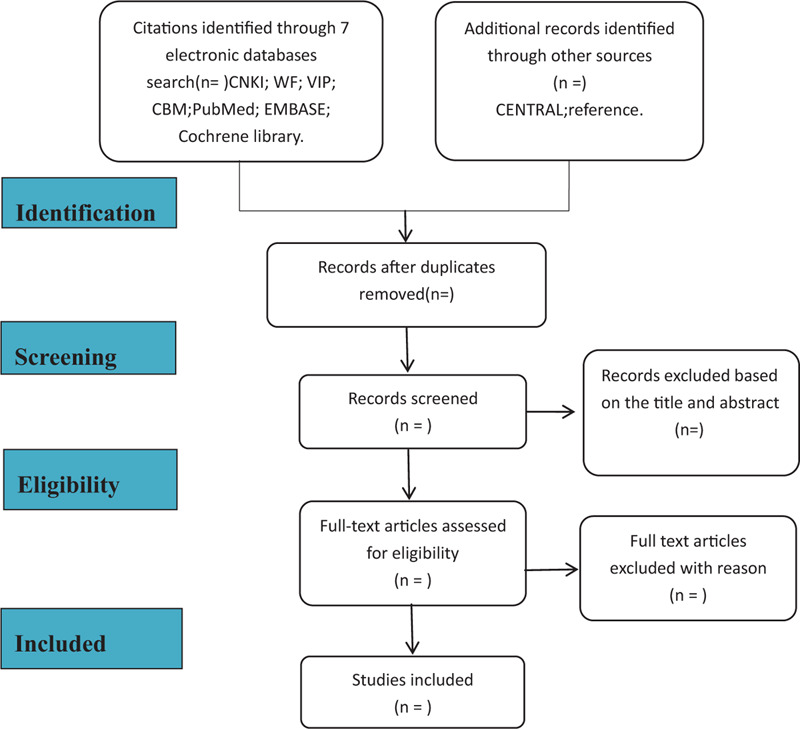
Flowchart of literature selection.

Two researchers (J and L) will retrieve all the documents we need in the database based on the correct subject terms, import these documents into the document manager note express 3.0, and delete the duplicate documents. Then read the titles and abstracts of the remaining documents, and delete the documents irrelevant to this systematic review. If the reading of the abstract and title cannot determine whether the document meets the standard, the researcher will read the full text of the article before making a judgment. Finally, download the remaining articles one by one to read the full text, and then determine the final required documents according to the various standards discussed above. In this process, the 2 researchers need to operate completely independently and strictly follow the operating procedures of the systematic review. If 2 people have ambiguity about the same article, ask the third reviewer (L) to negotiate. The specific process of the included literature can be seen in Figure 1.

#### 2.4.2 Data extraction and management

In the process of document inclusion this time, the required inclusion information was separately extracted by 2 researchers separately and then entered into Excel 2010. When the information extracted by the 2 researchers was entered, they were cross-checked and checked. If there is ambiguity in the inclusion of the literature, you can discuss with the third party (L) to ensure the credibility of the included article. The included information mainly includes the following aspects: the author of the article, the title of the document, the sample size, and the outcome indicators. If the important information that needs to be included in the article is missing, we will contact the author of the article by phone or email to ensure the authenticity of the information. If the author cannot be contacted or the required information is not available, we will give up this information.

### 2.5 Methodological evaluation

The method of article quality evaluation and bias risk assessment was done through Cochrane Reviewer's Handbook 5.0.^[26]^ Its main content includes the following 7 items: random method selection; allocation concealment; blinding; completeness of outcome data; whether the rater is blinded; selective reporting of results; other biases, all of the above 7 items contain “yes” and “no.” As well as the 3 options of “unclear,” 2 evaluators should make appropriate evaluations among the options. If 2 researchers disagree during the selection process, they need to consult a third party (L) for handling.

### 2.6 Data synthesis

#### 2.6.1 Measures of treatment effect

Two researchers (J and L) will use Review Manager software and Stata software for statistical analysis, and then synthesize all data. For categorical data, we will use risk ratios and 95% confidence intervals (CIs) to calculate and summarize the data. For continuous data, the mean difference and 95% confidence intervals (95% CIs) will be used to represent the result of data synthesis. If the outcome variables of different measurement scales are measured, a standardized mean difference analysis with 95% CI is performed.

#### 2.6.2 Management of missing data

In the research process, if there is data loss or incomplete data in the literature that affects the next step of the research, we will contact the author by email or phone to obtain the data we need. If the author cannot be contacted, we will follow the Cochrane Handbook methods and estimate the missing means and standard deviations of the baseline change based on existing baseline data and other data.^[27]^ If none of the above methods can get the data we need, we will consider giving up this information.

#### 2.6.3 Describe the heterogeneity of the data

In the research process, we will use I^2^ to test the heterogeneity. For the case of using the solid effect model, it is in line with *P*>.1 and I^2^<50%; when using the random effect model, it is in line with the case of *P*<.1 and I^2^>50%. If substantial heterogeneity is found in the analysis process, descriptive analysis can be used.

#### 2.6.4 Assessment of reporting biases

When more than 10 randomized controlled trials are included in the study, we will use a funnel chart to assess publication bias. In addition, we will use the Egger test to explore the potential causes of publication bias when the funnel chart is asymmetric.

#### 2.6.5 Subgroup analysis

If there is a large heterogeneity in the articles we included, we will conduct a subgroup analysis to reduce the clinical heterogeneity between the groups.

#### 2.6.6 Sensitivity analysis

To evaluate the true reliability of this systematic review and exclude low-quality experiments, we will use the software STATA 14.0 to perform sensitivity analysis if the data is sufficient.

#### 2.6.7 Grading the quality of evidence

During the research process, the 2 researchers will use the recommended evaluation and development evaluation grading system to independently evaluate the quality of evidence for all research results. Then, in accordance with the relevant rating standards, 4 levels of “high,” “medium,” “low,” and “very low” are adopted to rate the quality of research evidence.^[28,29]^

#### 2.6.8 Ethics and dissemination

This research is based on the literature, there is no need to recruit patients, nor contact with patients, and there is no need to collect patients’ personal information. Therefore, this study does not require ethical approval. This research will be published in a peer-reviewed journal to provide new ideas and methods for clinical treatment.

## 3 Discussion

Obesity has become one of the biggest chronic diseases in the world today accompanied by various life-threatening complications. Therefore, for all countries in the world, it is urgent to solve the obesity problem. There are a wide variety of current treatments for obesity. As a complementary and alternative therapy, Tuina has gradually revealed its advantages. At present, there are many kinds of research on the treatment of obesity by massage. However, the research on the effectiveness and safety of massage-related therapies in the treatment of various types of obesity in adults is still in a blank state. Therefore, we hope that through this research, we can comprehensively evaluate its effectiveness and safety, and provide new ideas and methods for clinical treatment of diseases.

## Author contributions

**Conceptualization:** Mengke Jin, Wei Xu.

**Data curation:** Xuefang Liu.

**Funding acquisition:** Lin Jiao.

**Methodology:** Jun Li.

**Software:** Daocheng Zhu, Genping Zhong.

**Writing – original draft:** Zhiwen Cao.

**Writing – review & editing:** Lin Jiao.
